# Feature Extraction of Ship-Radiated Noise Based on Intrinsic Time-Scale Decomposition and a Statistical Complexity Measure

**DOI:** 10.3390/e21111079

**Published:** 2019-11-04

**Authors:** Junxiong Wang, Zhe Chen

**Affiliations:** School of Marine Science and Technology, Northwestern Polytechnical University, Xi’an 710072, China; chenzhe@mail.nwpu.edu.cn

**Keywords:** statistical complexity measure, complexity-spectrum entropy plane, intrinsic time-scale decomposition, feature extraction

## Abstract

Extracting effective features from ship-radiated noise is an important way to improve the detection and recognition performance of passive sonar. Complexity features of ship-radiated noise have attracted increasing amounts of attention. However, the traditional definition of complexity based on entropy (information stored in the system) is not accurate. To this end, a new statistical complexity measure is proposed in this paper based on spectrum entropy and disequilibrium. Since the spectrum features are unique to the class of the ship, our method can distinguish different ships according to their location in the two-dimensional plane composed of complexity and spectrum entropy (CSEP). To weaken the influence of ocean ambient noise, the intrinsic time-scale decomposition (ITD) is applied to preprocess the data in this study. The effectiveness of the proposed method is validated through a classification experiment of four types of marine vessels. The recognition rate of the ITD-CSEP methodology achieved 94%, which is much higher than that of traditional feature extraction methods. Moreover, the ITD-CSEP is fast and parameter free. Hence, the method can be applied in the real time processing practical applications.

## 1. Introduction

Feature extraction of ship-radiated noise has attracted considerable attention from sonar engineers because it is an important way to improve the detection and recognition performance of passive sonar [1,2,3,4,5,6]. 

Due to the time-varying nature of the ocean medium and the interference of ocean ambient noise, it is challenging to extract effective characteristics from the received ship sound [7]. Traditional feature extraction methods, including the power spectrum density (PSD) and the wavelet transform, attempt to solve this problem in the frequency domain [7,8,9]. In general, the spectrum of a ship-radiated noise contains the continuous broadband spectral envelope and the narrowband discrete tones (i.e., the spectral lines). For a long time, the line-spectrum features have been widely used for ship detection and classification. This is because the narrowband spectral lines are produced by the rotating machinery (e.g., pumps, propellers, and engines) that are unique to the class of the vessel [10,11]. Since PSD is not able to reflect the time-varying characteristics of the signal to be analyzed, the wavelet transform, which can provide time–frequency information simultaneously, has received much attention [12,13]. However, there is still the lack of criteria to select the wavelet basis function that may influence the performance of the wavelet transform [6,9]. In addition, the well-known line-spectrum shift phenomenon limits the performance of spectrum-based methods [2]. 

Entropy is one of the most powerful metrics to evaluate the disorder degree (randomness) and unpredictability of a signal [14]. There have been a lot of entropic algorithms in the literature, such as permutation entropy (PE) [15], sample entropy (SE) [16], fuzzy entropy [17], dispersion entropy (DE) [18], and fluctuation-based dispersion entropy (FDE) [19]. These methodologies are applicable for any type of signal, whether they are linear or nonlinear, stationary or nonstationary, chaotic or stochastic. Owing to this, they have attracted increasing attention in the field of underwater acoustic signal processing. Shashidhar et al. applied SE to detect a weak target embedded in ocean ambient noise [20]. Chen et al. [21] proposed an improved version of PE to classify different types of marine vessels under noisy conditions. In order to weaken the influence of ocean noise, the de-noising procedure has been performed prior to the entropy estimation in recent studies, utilizing the famous variational mode decomposition (VMD), empirical mode decomposition (EMD), and its modifications [22,23,24,25]. To process the data in real time, a recent work used intrinsic time-scale decomposition (ITD) instead of EMD and VMD [26,27]. 

The definition of entropy is associated with the information stored in the system, and to some extent, it can reflect the complexity of the physical process. However, it is crude to simply defines the complexity in terms of only order and information [28,29]. For example, despite its unpredictable and disordered character, a white-noise random process should also be regarded as simple since it does not contain any non-trivial structure [30,31]. In order to comprehensively define the complexity, a statistical complexity measure called Lopez–Mancini–Calbert (LMC) complexity measure was proposed in Ricardo et al. [29] by integrating the entropy *H* and disequilibrium *Q* (i.e., complexity *C* = *HQ*), where the disequilibrium denotes the distance between the given probability distribution and the equilibrium distribution of the accessible states of the system [29,30,31] (for a detailed description of the method, please check Section 2.2). By this definition, a given value of *H* can correspond to a range of possible *C* values. The two-dimensional plane composed of *H* and *C* is called the complexity–entropy plane, which can provide abundant information related to the correlation structure of the system [32]. It is worth noting that this plane is a valuable tool for differentiating time series. As a matter of fact, it has achieved great success in distinguishing between chaotic time series and stochastic ones [33,34,35].

**Figure 1:**
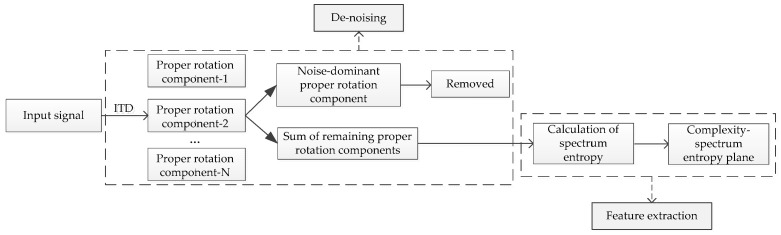
The flow diagram of feature extraction.

For the purpose of extracting complexity features from ship-radiated noise, in this paper, a new kind of statistical complexity measure is proposed based on spectrum entropy and disequilibrium. The flow diagram of the proposed feature extraction algorithm is depicted in Figure 1. First, ITD is applied to decompose the analyzed signal into some proper rotation components (PRCs), and the noise-dominant components are removed. After that, the sum of the remaining PRCs is transformed to the frequency domain. Then, the spectrum entropy is defined and the corresponding complexity is calculated. Since the spectrum features are unique to the class of the ship, our method can distinguish different types of ships according to their location in the two-dimensional plane composed of complexity and spectrum entropy (CSEP). Moreover, unlike the line spectrum that only considers the local character of the spectrum, our proposed method can adequately reflect the energy distribution structure of the spectrum. As will be shown below, the proposed ITD-CSEP methodology is fast, free of parameters, and can significantly improve the classification accuracy of diverse types of ships. 

The remainder of this paper is organized as follows: the basic theory is described in Section 2, feature extraction results are provided in Section 3, ship classification results are given in Section 4, and the paper is concluded in Section 5.

## 2. Basic Theory 

### 2.1. Intrinsic Time-Scale Decomposition (ITD)

ITD is a relatively new signal decomposition technique proposed by Frei et al. [27]. Compared with EMD and VMD, it can accurately extract the PRCs within a few iterations and thus is able to process data in real time. Thanks to this, we selected ITD for de-noising in our study. 

For a time series $X_{t}$(t≥0), let L(⋅) and H(⋅) be the baseline extraction operator and the PRC extraction operator, respectively. The relationship of $X_{t}$, L(⋅), and H(⋅) can be expressed as $X_{t}=L(X_{t})+(1-L)(X_{t})=L(X_{t})+H(X_{t})=L_{t}+H_{t}$. The main steps of the ITD algorithm are as follows:

1. Let $X_{k}$ be the local extrema of $X_{t}$ at time index $\tau_{k}$. Suppose that $X_{t}$ is available on $t\in (0,\tau_{k+2})$ and that $L_{t}$ is defined on interval $\left[0,\tau_{k}\right]$. Then, $L_{t}$ on the interval $\left(\tau_{k},\tau_{k+1}\right]$ can be computed using:
  (1) $$L(X_{t})=L_{t}=L_{k}+(\frac{L_{k+1}-L_{k}}{X_{k+1}-X_{k}})(X_{t}-X_{k}),\;t\in (\tau_{k},\tau_{k+1}]$$
  where:
  (2) $$L_{k+1}=\alpha \left[X_{k}+(\frac{\tau_{k+1}-\tau_{k}}{\tau_{k+2}-\tau_{k}})(X_{k+2}-X_{k})\right]+(1-\alpha )X_{k+1}$$
  According to Frei and Osorio [27], the constant α is typically fixed at α=0.5.
2. Set the obtained $L_{t}$ as the input signal and continue the iteration until the terminal condition is reached. In our study, once the energy of $L_{t}$ was less than 1% of $X_{t}$, the iteration was stopped.
3. Finally, the ITD of $X_{t}$ can be expressed as:
  (3) $$\begin{array}{l} X_{t}=H(X_{t})+L(X_{t})=H(X_{t})+(H+L)L(X_{t})=(H(1+L)+L^{2})(X_{t})\\ =(H\sum_{k=0}^{P-1}L^{k}+L^{p})(X_{t}) \end{array}$$
  where $HL^{k}(X_{t})$ denotes the obtained PRC after *k +* 1 iterations, and $L^{p}(X_{t})$ is the monotonic trend (if the terminal condition is reached before the monotonic trend is obtained, $L^{p}(X_{t})$ represents the lowest frequency baseline). ITD is a fully data-driven method; the produced PRCs are adaptively arranged in order from high frequency to low frequency in the frequency domain. In general, the *k*th PRC will be “noisier” than that of the (*k* + 1)th [26,36]. Hence, in this study, the first PRC of the ship-radiated noise is regarded as a noise-dominant component, and is removed.

### 2.2. LMC Complexity Measure

In order to comprehensively define the complexity, a statistical complexity measure called the Lopez–Mancini–Calbert (LMC) complexity measure was proposed in Ricardo et al. [29]. First, LMC defines the disequilibrium Q[P] as:

(4) $$Q[P]=Q_{0}\cdot D[P,P_{e}]$$

In Equation (4),$Q_{0}$ is a normalization constant ranging from 0 to 1; $P=\left\{p_{j},j=1,\ldots ,N\right\}$ denotes the probability distribution of the system; $P_{e}$ represents the equilibrium distribution; and $D[P,P_{e}]$ is the distance between P and $P_{e}$. Then, the LMC complexity measure, which combines the concepts of entropy and disequilibrium, is defined as:

(5) C[P]=H[P]⋅Q[P]

where H[P] stands for the entropy of the system. By this definition, a given value of H[P] may correspond to a range of possible C[P] values. The two-dimensional plane composed of H[P] and C[P] is called the complexity–entropy plane, which can provide abundant information related to the correlation structure of the system [32].

### 2.3. Complexity–Spectrum Entropy Plane

There are several approaches toward quantifying H[P]. Without being exhaustive, we can enumerate Shannon entropy, Tsallis entropy, Renyi entropy, and permutation entropy [28,29,30,31,32]. There are also a variety of distance metrics used to compute the disequilibrium Q[P], including the Euclidean norm, the Wootters’s distance, and the Jensen divergence. In this paper, a new statistical complexity measure is proposed. The spectrum entropy is applied to calculate H[P], and the Jensen divergence is selected to measure Q[P]. For a time-series $x=\{x_{1},x_{2},\ldots ,x_{N}\}$, the proposed CSEP is calculated as follows:

1. Transform the input signal to the frequency domain using:
  (6) $$P(k)=\frac{{\left|X(k)\right|}^{2}}{N_{fft}},k=1,2\ldots ,N_{fft}$$
  where X is the Fourier transform of x, X(k) is a frequency point of X, and $N_{fft}$ is the length of X.
2. The probability distribution of X can be computed using:
  (7) $$p_{k}=\frac{X(k)}{\sum_{k=1}^{N_{fft}}X(k)},k=1,2\ldots ,N_{fft}$$
3. The spectrum entropy and its normalized version are then, respectively, defined as:
  (8) $$H_{SPE}=-\sum_{k=1}^{N_{fft}}p_{k}\cdot \log(p_{k})$$
  (9) $$H_{NSPE}=H_{SPE}/-\sum_{k=1}^{N_{fft}}p_{e}\cdot \log(p_{e})=H_{SPE}/\log(N_{fft}),\;p_{e}=1/N_{fft}$$
4. Compute the disequilibrium $Q_{SPE}$ using Equations (10)–(12), where the distance between ${\left\{p_{k}\right\}}_{1}^{N_{fft}}$ and ${\left\{p_{e}\right\}}_{1}^{N_{fft}}$ are calculated using the Jensen divergence:
  (10) $$Q_{0}=-2{\left\{(\frac{N_{fft}+1}{N_{fft}})\cdot \log(N_{fft}+1)-2\cdot \log(2\cdot N_{fft})+\log(N_{fft})\right\}}^{-1}$$
  (11) $$J_{SPE}=-\sum_{k=1}^{N_{fft}}\left(\frac{p_{k}+p_{e}}{2}\right)\cdot \log(\frac{p_{k}+p_{e}}{2})-0.5\cdot H_{SPE}-0.5\cdot \log(N_{fft})$$
  (12) $$Q_{SPE}=Q_{0}\cdot J_{SPE}$$
5. Define the new complexity $C_{SPE}$ as:
  (13) $$C_{SPE}=Q_{SPE}\cdot H_{NSPE}$$
6. Finally, the two-dimensional plane composed of $H_{NSPE}$ and $C_{SPE}$ is called the CSEP, which can be used to discriminate different types of ship-radiated noise according to their location (i.e., the ($H_{NSPE}$,$C_{SPE}$) points).

## 3. Results and Discussion

In this section, the effectiveness of the proposed method is validated through analyzing four types of real ship-radiated noise. 

### 3.1. Data Description

**Figure 2:**
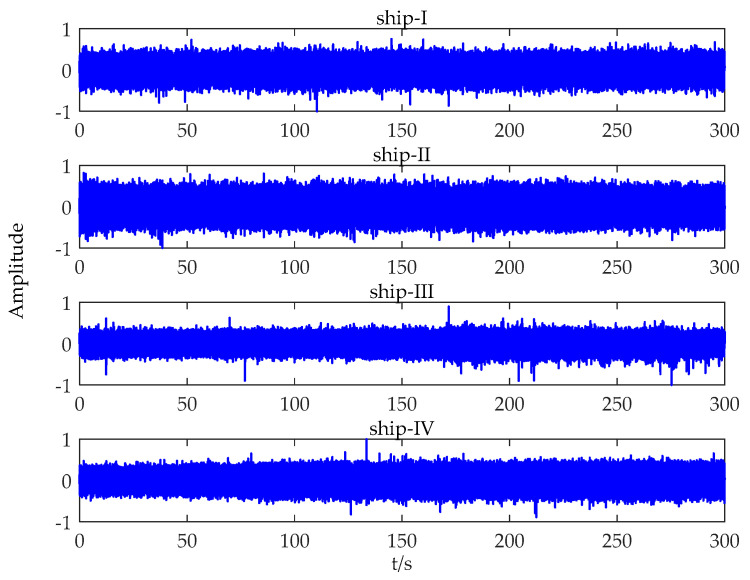
The time domain waveforms of four kinds of marine vessels where ships I–IV are a cruise ship, freighter, ocean liner, and oiler, respectively.

**Figure 3:**
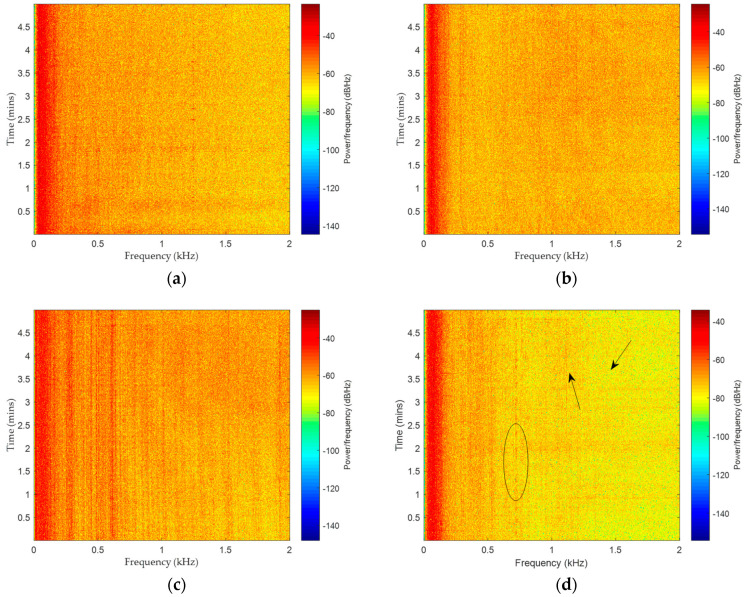
Spectrograms of four kinds of marine vessels: (**a**) ship-I, (**b**) ship-II, (**c**) ship-III, and (**d**) ship-IV.

The data used in this study were measured in South China Sea, containing four types of marine vessels: cruise ship, freighter, ocean liner, and oiler (the four types of ships are denoted as ship-I, ship-II, ship-III, and ship-IV, respectively). An omnidirectional hydrophone, whose sampling rate is 20 kHz, was mounted at a depth of 30 m. The ship-radiated noise was recorded when different kinds of vessels passed by the hydrophone within a range of ≈500–1500 m. It is worth mentioning that only one vessel was measured for each type of ship. The time domain waveforms of diverse ship-radiated noise are provided in Figure 2, and the corresponding spectrograms are offered in Figure 3.

The line-spectrum features are obvious in Figure 3a–d. In practical engineering, ship recognition is usually done according to the frequency of spectral lines. However, it is found that the line-spectrum features are not so stable. Taking Figure 3d as an example, at the point where the arrows directed, there are two spectral lines that somehow appear suddenly. In addition, within the region of the ellipse, the spectral line at 724 Hz sometimes disappears. The unstable line-spectrum features might cause the ship recognition rate to decline. 

### 3.2. Complexity Feature Extraction of Ship-Radiated Noise

In this subsection, the experimental data were analyzed using our methodology (ITD-CSEP). For comparison purposes, the data were also processed using the multi-scale dispersion entropy (MDE) [19]. Each type of ship-radiated noise contained 6 million sample points (i.e., duration of 300 s), which was equally cut into 300 pieces.

**Figure 4:**
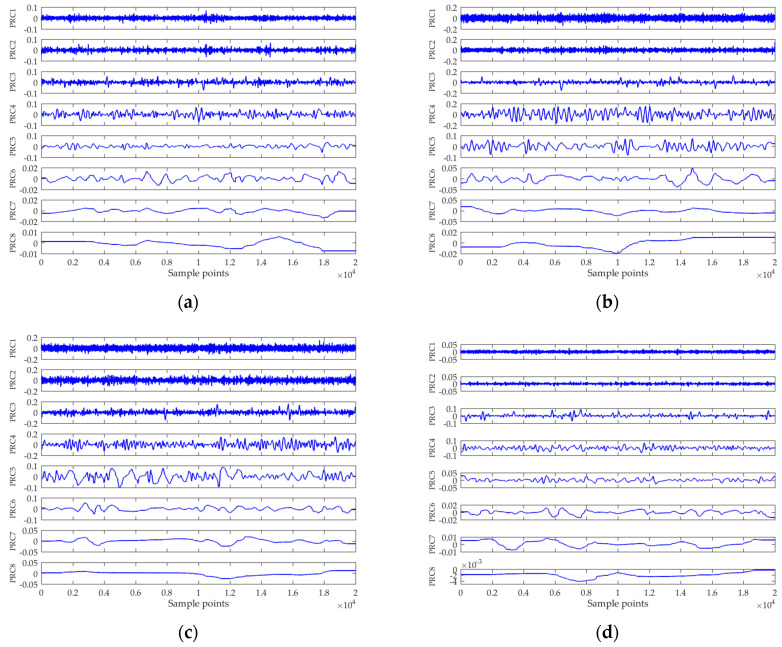
ITD results of four kinds of marine vessels: (**a**) ship-I, (**b**) ship-II, (**c**) ship-III, and (**d**) ship-IV.

The ITD results are depicted in Figure 4. For each type of ship, only one piece of data is randomly selected to show. As can be seen, the PRCs are arranged in order from high frequency to low frequency. In general, the *k*th PRC will be “noisier” than that of the (*k* + 1)th [26,36]. Hence, the first PRC is regarded as a noise-dominant component, and is removed. Compared with VMD, ITD can extract the PRCs accurately within a few iterations and thus is able to process data in real time. Table 1 compares the running time of VMD and ITD for processing 10 pieces of data (parameters for running the VMD algorithm were set to be the same according to Yang et al. [24]). Both algorithms were run on a PC with an Intel(R) Core(TM) i5-7300HQ CPU at 2.50 GHz with the MATLAB (R2016a, Mathworks, Natick, MA, United States of America) platform. It was found that VMD demanded much more computation time than that of ITD, which was unacceptable for our real-time processing application.

**Figure 5:**
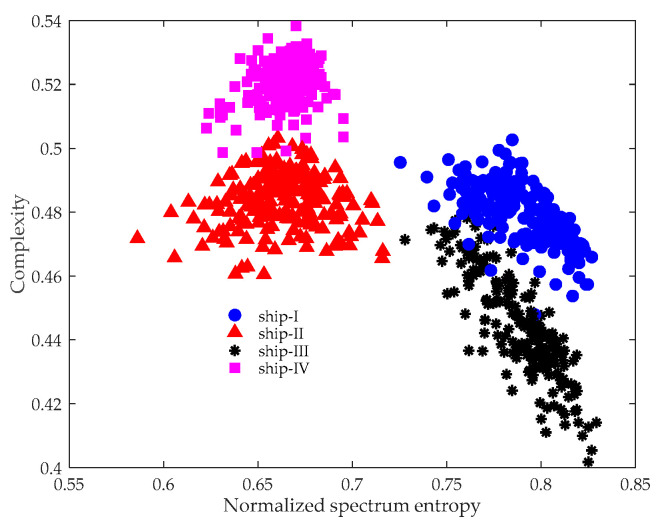
ITD-CSEP results of four kinds of marine vessels.

After de-noising, the sum of the remaining PRCs was processed using the CSEP. The results are plotted in Figure 5, where diverse ships are represented with different color and symbols. It can be seen that different kinds of vessels are located in distinct regions in the CSEP, illustrating that the method is effective for ship classification. Unlike a line spectrum that only considers the local character of the spectrum, the proposed method can adequately reflect the energy distribution structure of the spectrum. Since spectrum features are unique to the class of the ship, the effectiveness of our method is reasonable. 

**Figure 6:**
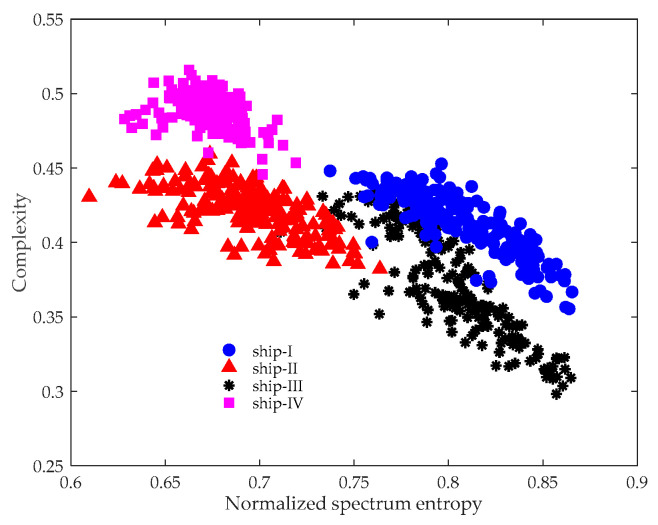
CSEP results of four kinds of marine vessels.

It is also important to show why the experimental data is not processed directly using the CSEP. Figure 6 provides the CSEP results without preprocessing using ITD. In contrast to Figure 5, the distance between the features obviously became closer, meaning it was more difficult to discriminate between the targets. Even though the data analyzed in our study was measured at a close distance, they were inevitably contaminated by ocean ambient noise. Hence, applying ITD to de-noise is of great necessity.

**Figure 7:**
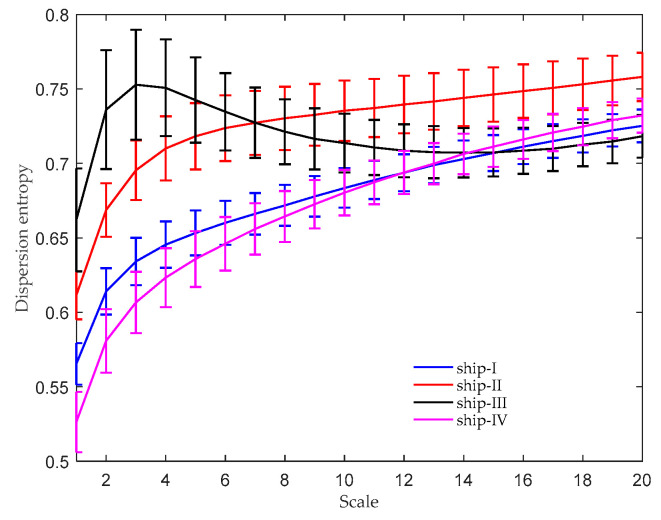
MDE results of four kinds of marine vessels.

For comparison purposes, the MDE [19] and selective noise-assisted EMD (SN-EMD) [25] were also utilized to extract features of the four types of ships. There were several parameters that needed to be predefined in MDE, including the embedding dimension m, time delay τ, classes c, and scale factor s. According to the suggestion in Azami and Escudero [19], they were set as m=4, τ=1, c=6, and s=20. The MDE results are shown in Figure 7, where the mean DE values with their standard deviation (SD) error bars are plotted. As can be seen, the DE values of ship-I and ship-IV overlapped with each other over most scales, especially when s≥10. It is worth noting that the performance of MDE may be quite different if the parameters are not appropriately selected.

**Figure 8:**
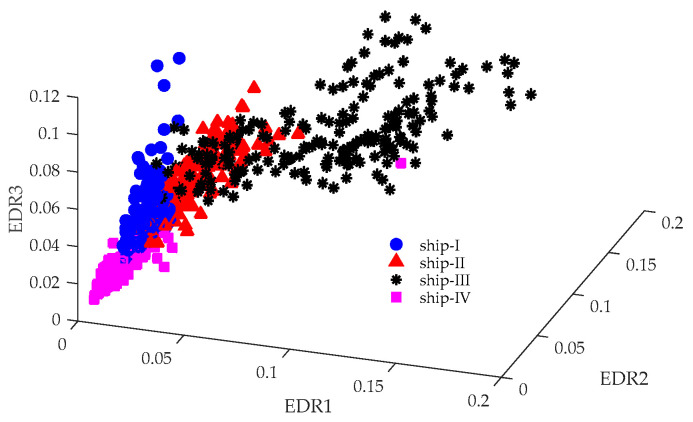
SN-EMD-EDR results of four kinds of marine vessels.

The SN-EMD [25] is a modification of the EMD, which overcomes the mode mixing problem. As a consequence, it obtains more accurate decomposition results. The energy distribution ratio (EDR) of each intrinsic mode function (IMF) is one of the most effective features for ship recognition [25]. For a fair comparison, parameters for computing the SN-EMD were set to be the same as those in Niu et al. [25]. According to Niu et al. [25], the EDR of the first three IMFs are plotted in Figure 8, where $EDR_{i}$ refers to the EDR of the *i*th IMF. It is seen that, despite some overlapping, different targets are located in distinct regions in the three-dimensional space.

## 4. Pattern Recognition

To evaluate the feature extraction performance quantitatively, the probability neural network (PNN) [37] was applied to further process the extracted features. For each type of ship, 200 randomly selected pieces of data were used for training and the remaining 100 pieces were used for testing. Table 2, Table 3, Table 4, Table 5 and Table 6 demonstrate the detailed classification results of the test data, which correspond well with the feature extraction results in Section 3. The ITD-CSEP obtained the highest classification accuracy of 94%, followed by MDE, CSEP, SN-EMD-EDR, and PSD, with recognition accuracies of 87.75%, 83.5%, 83%, and 68.25%, respectively. The pattern recognition results further proved the effectiveness of the proposed method for the feature extraction of ship-radiated noise.

Since our application requires real time processing, it is also necessary to compare the computation cost of above-mentioned feature extraction methods. Table 7 lists the computation time these methods needed to process all 1200 pieces of data. All algorithms were run on a PC with an Intel(R) Core(TM) i5-7300HQ CPU at 2.50 GHz with the MATLAB (R2016a, Mathworks, Natick, MA, United States of America) platform. It can be found that PSD and ITD-CSEP ran significantly faster than other algorithms. Generally, the ITD-CSEP obtained the highest recognition rate with an acceptable computation cost.

## 5. Conclusions

In order to extract effective features from ship-radiated noise, a new statistical complexity measure is proposed in this paper based on spectrum entropy and disequilibrium. Since the spectrum features are unique to the class of the ship, the proposed method can distinguish different types of ships according to their location in the two-dimensional CSEP. In order to weaken the influence of ocean noise, the intrinsic time-scale decomposition (ITD) was utilized for de-noising in this study. Advantages of the ITD-CSEP methodology are listed below:

1. The proposed algorithm was fast. It only required 81.82 s to process all 1200 pieces of data while the MDE and SN-EMD-EDR needed 528.27 s (scale = 1–20) and 825.6 s, respectively.
2. Unlike MDE and VMD whose performance may be influenced by parameter selection, the ITD-CSEP is completely free of parameters.
3. The ITD-CSEP features are unique for different types of ships. The ship classification experiment proves that the recognition rate of the proposed method achieved 94%, which was much higher than other traditional feature extraction methods.

Owing to the above-mentioned advantages of the proposed methodology, the ITD-CSEP algorithm is suitable for our practical application that requires real-time processing. In future studies, our methodology can be extended for image de-noising.

## Figures and Tables

**Table 1:** Comparison of VMD and ITD in terms of computation time for processing 10 pieces of data.

	ITD	VMD
Computation time	0.8 s	557.8 s

**Table 2:** PNN classification results of ITD-CSEP.

Type	Recognized as				Accuracy
	Ship-I	Ship-II	Ship-III	Ship-IV	
Ship-I	83	6	4	7	83%
Ship-II	0	99	1	0	99%
Ship-III	4	0	96	0	96%
Ship-IV	0	2	0	98	98%
In total	-	-	-	-	94%

**Table 3:** PNN classification results of CSEP.

Type	Recognized as				Accuracy
	Ship-I	Ship-II	Ship-III	Ship-IV	
Ship-I	74	0	26	0	74%
Ship-II	0	97	2	1	97%
Ship-III	34	1	65	0	65%
Ship-IV	0	2	0	98	98%
In total	-	-	-	-	83.5%

**Table 4:** PNN classification results of MDE.

Type	Recognized as				Accuracy
	Ship-I	Ship-II	Ship-III	Ship-IV	
Ship-I	96	3	0	1	96%
Ship-II	10	82	8	0	82%
Ship-III	0	0	100	0	100%
Ship-IV	26	1	0	73	73%
In total	-	-	-	-	87.75%

**Table 5:** PNN classification results of SN-EMD-EDR.

Type	Recognized as				Accuracy
	Ship-I	Ship-II	Ship-III	Ship-IV	
Ship-I	83	13	4	0	83%
Ship-II	6	58	36	0	58%
Ship-III	0	0	100	0	100%
Ship-IV	8	0	1	91	91%
In total	-	-	-	-	83%

**Table 6:** PNN classification results of PSD.

Type	Recognized as				Accuracy
	Ship-I	Ship-II	Ship-III	Ship-IV	
Ship-I	61	0	9	30	61%
Ship-II	0	99	1	0	99%
Ship-III	0	48	52	0	52%
Ship-IV	38	0	1	61	61%
In total	-	-	-	-	68.25%

**Table 7:** Comparison of feature extraction methods in terms of computation time for analyzing 1200 pieces of data.

	ITD-CSEP	MDE (scale = 1–20)	MDE (scale = 1–10)	SN-EMD-EDR	PSD
Computation time	81.82 s	528.27 s	390.87 s	825.6 s	3.19 s
